# Dopamine regulates pancreatic glucagon and insulin secretion via adrenergic and dopaminergic receptors

**DOI:** 10.1038/s41398-020-01171-z

**Published:** 2021-02-16

**Authors:** Despoina Aslanoglou, Suzanne Bertera, Marta Sánchez-Soto, R. Benjamin Free, Jeongkyung Lee, Wei Zong, Xiangning Xue, Shristi Shrestha, Marcela Brissova, Ryan W. Logan, Claes B. Wollheim, Massimo Trucco, Vijay K. Yechoor, David R. Sibley, Rita Bottino, Zachary Freyberg

**Affiliations:** 1 Translational Neuroscience Program, Department of Psychiatry, University of Pittsburgh, Pittsburgh, PA USA; 2 Institute of Cellular Therapeutics, Allegheny Health Network Research Institute, Allegheny Health Network, Pittsburgh, PA USA; 3 Molecular Neuropharmacology Section, National Institute of Neurological Disorders and Stroke, National Institutes of Health, Bethesda, MD USA; 4 Division of Endocrinology, Diabetes & Metabolism, Department of Medicine, Diabetes and Beta Cell Biology Center, University of Pittsburgh, Pittsburgh, PA USA; 5 Department of Biostatistics, University of Pittsburgh, Pittsburgh, PA USA; 6 Division of Diabetes, Endocrinology, and Metabolism, Department of Medicine, Vanderbilt University Medical Center, Nashville, TN USA; 7 Center for Systems Neurogenetics of Addiction, The Jackson Laboratory, Bar Harbor, ME USA; 8 Department of Cell Physiology and Metabolism, University of Geneva, Geneva, Switzerland; 9 Department of Biological Sciences, Carnegie Mellon University, Pittsburgh, PA USA; 10 College of Medicine, Drexel University, Philadelphia, PA USA; 11 Department of Cell Biology, University of Pittsburgh, Pittsburgh, PA USA

**Keywords:** Physiology, Pathogenesis

## Abstract

Dopamine (DA) and norepinephrine (NE) are catecholamines primarily studied in the central nervous system that also act in the pancreas as peripheral regulators of metabolism. Pancreatic catecholamine signaling has also been increasingly implicated as a mechanism responsible for the metabolic disturbances produced by antipsychotic drugs (APDs). Critically, however, the mechanisms by which catecholamines modulate pancreatic hormone release are not completely understood. We show that human and mouse pancreatic α- and β-cells express the catecholamine biosynthetic and signaling machinery, and that α-cells synthesize DA de novo. This locally-produced pancreatic DA signals via both α- and β-cell adrenergic and dopaminergic receptors with different affinities to regulate glucagon and insulin release. Significantly, we show DA functions as a biased agonist at α_2A_-adrenergic receptors, preferentially signaling via the canonical G protein-mediated pathway. Our findings highlight the interplay between DA and NE signaling as a novel form of regulation to modulate pancreatic hormone release. Lastly, pharmacological blockade of DA D_2_-like receptors in human islets with APDs significantly raises insulin and glucagon release. This offers a new mechanism where APDs act directly on islet α- and β-cell targets to produce metabolic disturbances.

## Introduction

Antipsychotic drugs (APDs) are widely used worldwide to treat highly prevalent psychiatric illnesses including schizophrenia, bipolar disorder, and major depressive disorder^1,2^. However, these drugs also cause profound metabolic dysfunction including dysglycemia and systemic insulin resistance that increase the risk of type 2 diabetes (T2D)^3^. Significantly, all APDs cause metabolic side effects to differing degrees, and current treatments to reduce these metabolic symptoms have only limited efficacy^4^. The single unifying property of APDs is their blockade of dopamine D_2_-like receptors, including D_2_ (D2R) and D_3_ (D3R) receptors. This suggests key roles for D2R and D3R not only in promoting APDs’ therapeutic actions, but also in causing their metabolic side effects.

We and our colleagues found that D2R and D3R are expressed not only in the central nervous system (CNS) but also in pancreatic β-cells, and that these receptors are important negative regulators of insulin secretion^5–9^. Direct stimulation of β-cell D2R and D3R with dopamine (DA) or D2R/D3R agonists inhibits glucose-stimulated insulin secretion (GSIS) in both human and mouse pancreatic islets^6–8,10^, suggesting that β-cell D2R/D3R signaling is an important regulator of GSIS^5,6,8,9^. Indeed, in vivo human and rodent studies demonstrated that treatment with the DA precursor L-DOPA causes hyperglycemia due to decreased GSIS^11–14^. Conversely, we previously showed that the APD blockade of β-cell D2R/D3R enhances GSIS^6^. As in T2D, APD-induced increases in circulating insulin are hypothesized to desensitize insulin-sensitive peripheral targets (e.g., liver, skeletal muscle, adipose tissue) over time, resulting in insulin resistance and weight gain^3,4,15^.

Despite a still-limited understanding of dopaminergic regulation of β-cells, even less is known about glucagon-secreting pancreatic α-cells^16^. Like insulin, glucagon is a key regulator of glucose homeostasis, raising blood glucose during decreased glucose availability via stimulation of hepatic glucose production^17,18^. Notably, inappropriately elevated glucagon levels are a characteristic feature of APD treatment in humans and rodents^16,19–21^. Since rodent and human α-cells express DA D_2_-like receptors^22,23^, APDs may also directly target α-cell D2R/D3R to raise glucagon and drive hyperglycemia, further contributing to insulin resistance. However, little is known about the roles of these DA receptors in regulating α-cell function. Thus, elucidating D2R/D3R signaling in α-cells may shed important new light on dopaminergic regulation of glucagon release as well as offer new mechanisms and therapeutic targets for APD-induced metabolic disturbances.

Just as fundamentally, the precise sources of DA acting on α- and β-cell dopaminergic receptors are still poorly understood. Earlier work primarily focused on islet sources of the related catecholamine, norepinephrine (NE)^6,7,9,24^. Sympathetic nervous innervation was long considered a primary source of NE acting on the endocrine pancreas^25,26^. However, the degree of islet innervation is unclear, particularly in humans, and recent evidence suggests only sparse sympathetic innervation of human islets^26–32^. Islets also lack direct dopaminergic innervation^32,33^. Together, these data point to the importance of locally-produced, non-neuronal sources of both NE and DA for catecholamine signaling in α- and β-cells^6,7,32,33^. Indeed, mouse and human islets produce DA independently of sympathetic innervation, particularly in response to uptake of precursors like l-3,4-dihydroxyphenylalanine (L-DOPA)^6,7,9,30,33–36^. We and others previously showed that human and rodent β-cells express critical components of the catecholamine biosynthetic machinery including tyrosine hydroxylase (TH), Dopa decarboxylase (DDC), and the vesicular monoamine transporter (VMAT), allowing these cells to produce, package and secrete DA^6,7,9,37–39^. Nevertheless, it remains unclear whether additional islet cell types including α-cells are also responsible for islet catecholamine production.

Here, we show that, like β-cells, mouse and human α-cells express the complete catecholamine biosynthetic, transport, and catabolic machinery. Consequently, α-cells synthesize both DA and NE de novo, though preferentially produce DA in response to uptake of precursor L-DOPA. Significantly, we provide evidence that DA modulates both glucagon and insulin release by signaling directly via α- and β-cell adrenergic receptors in human and mouse islets. Furthermore, at α_2A_-adrenergic receptors, DA functions as a biased ligand, preferentially signaling via Gα protein recruitment but not through β-arrestin2, providing a new mechanism for DA’s intracellular signaling at these receptors. Lastly, we demonstrate that pharmacological D_2_-like receptor blockade by APDs significantly elevates glucagon as well as insulin secretion in human pancreatic islets. This suggests that APDs act directly on islet α-cells, in addition to β-cells, to disrupt the D_2_-like receptor signaling responsible for regulating islet hormone secretion. Thus, such APD-induced disruptions may drive the development of disturbed glucose homeostasis commonly found in treated patients^16,40,41^. Together, our findings provide new mechanisms for how DA and NE signaling regulates insulin and glucagon release. This work also offers a new context for APDs’ actions in the periphery that may explain how APDs produce metabolic dysfunction.

## Materials and methods

### Compounds

Compounds used in this study were purchased from Sigma-Aldrich (St. Louis, MO) unless indicated otherwise: HEPES, sodium pyruvate (Gibco/ThermoFisher Scientific, Pittsburgh, PA), penicillin/streptomycin, 2-mercaptoethanol, D-glucose, bovine serum albumin (BSA; Merck Millipore, Darmstadt, Germany), EDTA, ascorbic acid, *S*-(−)-propranolol, *R*-(−)-deprenyl hydrochloride, pargyline hydrochloride, clorgyline hydrochloride, yohimbine (Tocris, Bristol, United Kingdom), haloperidol, clozapine, olanzapine (Tocris), and [^3^H]RX821002 (Perkin Elmer, Billerica, MA).

### Cell culture

αTC1 clone 6 (αTC1–6) cells [American Type Tissue Culture Collection (ATCC), #CRL-2934, Manassas, VA] were cultured in DMEM medium (Gibco) supplemented with 10% fetal bovine serum (FBS), 100 U/mL penicillin/streptomycin, 15 mM HEPES, 0.1 mM non-essential amino acids, 0.02% BSA, 15 mg/L sodium bicarbonate, 2 mg/L glucose. INS-1E cells (gift of Dr. Pierre Maechler, Université de Genève) and all clonally-derived cell lines were cultured as described previously^7,10,42^. Briefly, INS-1E cells were maintained in RPMI 1640 medium (Gibco) supplemented with 5% (v/v) heat-inactivated fetal bovine serum, 2 mM l-glutamine, 10 mM HEPES, 1 mM sodium pyruvate, 100 U/mL penicillin/streptomycin and 50 μM 2-mercaptoethanol. HEK-293 cells (ATCC, #CRL-1573) were cultured in DMEM medium (Gibco) supplemented with 10% FBS and 100 U/mL penicillin/streptomycin. Cell lines were maintained in a humidified 37 °C incubator with 5% CO_2_. All cell lines tested negative for mycoplasma contamination.

### Generation of α_2A_-adrenergic receptor knockout cell line

We employed a CRISPR-Cas9-mediated approach to delete endogenous α_2A_-adrenergic receptor expression in INS-1E cells. Since INS-1E cells are rat-derived, we used the mRNA sequence encoding rat α_2A_-adrenergic receptor as a template to generate guide RNA (gRNA) to disrupt the promoter region of the *Adra2a* gene (gRNA sequence: 5′-GCAGCCGGATGCCGGCAATA-3′, positions 57–76). We then generated a construct containing the *Adra2a* gRNA sequence along with cDNA sequences encoding Cas9 and GFP (pENTR-Adra2a-sgRNA-Cas9-GFP). 1–5 μg of the construct was subsequently transfected into low-passage INS-1E cells using Lipofectamine 3000 (ThermoFisher Scientific) according to manufacturer instructions. 24–48 h post-transfection, the transfected cells were identified and collected by fluorescence-activated cell sorting based on GFP fluorescence using a BD FACSAria II sorter (BD Biosciences, San Jose, CA) equipped with a GFP filter (530/30 nm). Single cells were sorted into 96-well plates in complete RPMI 1640 medium and allowed to recover for 72 h before the addition of fresh media. Upon recovery and expansion, a number of clonal cell lines were generated and initially screened for complete α_2_-adrenergic receptor knockout by qPCR followed by additional functional validation via insulin secretion and radioligand binding assays.

### Animal husbandry

Animals were housed and handled in accordance with appropriate NIH guidelines through the University of Pittsburgh Institutional Animal Care and Use Committee (Protocol # 19075490), which approved the study. We abided by all appropriate animal care guidelines including ARRIVE guidelines for reporting animal research. Mice were housed in cages with a 12:12 light:dark cycle and had access to food and water ad lib at all times unless indicated otherwise. Every effort was made to ameliorate animal suffering.

### Human subjects

Pancreata and islets from non-diabetic adult donors were obtained via a partnership with CORE (Center for Organ Recovery and Education). Donor demographic information is summarized in Supplementary Table S1. The University of Pittsburgh Institutional Review Board declared studies on de-identified human pancreatic specimens do not qualify as human subject research.

### Pancreatic islet preparation

For mouse pancreatic islet preparations, islets were obtained from 8 to 10-week-old wild-type BALB/c mice. Islets were freshly isolated via collagenase digestion of pancreata as described previously^43^. Human pancreatic islets were isolated via collagenase digestion and purified from four non-diabetic human donors (Supplementary Table S1) as described previously^44^. Following isolation and purification, human or mouse islets were plated at a density of 15 islets per well into 24-well tissue culture-treated plates. Islets were allowed to recover overnight, free-floating in RPMI 1640 complete media, supplemented with 10% FBS. The islets were used immediately after overnight recovery for hormone secretion assays.

### Catecholamine secretion and measurement

#### Catecholamine secretion assay

Cell-based catecholamine secretion assays were conducted as reported earlier^7^. Briefly, αTC1–6 cells were seeded into 24-well plates at an initial seeding density of 5 × 10^5^ cells/well in complete DMEM medium. On the experimental day, cells were washed twice and placed into KRB buffer (132.2 mM NaCl, 3.6 mM KCl, 5 mM NaHCO_3_, 0.5 mM NaH_2_PO_4_, 0.5 mM MgCl_2_, 1.5 mM CaCl_2_ and 0.001 g/mL BSA, pH 7.4) supplemented with 25 mM glucose (1 h, 37 °C, 5% CO_2_). Some samples were also supplemented with 10 μM L-DOPA to boost cell catecholamine production in the presence or absence of a monoamine oxidase inhibitor cocktail (10 μM: deprenyl, pargyline, clorgyline). At assay conclusion, supernatants were immediately collected from each sample and placed directly into cold HeGa solution (0.1 M glacial acetic acid, 0.1 mM EDTA, and 0.12% oxidized l-glutathione, pH 3.7) on ice to protect catecholamine content from oxidation. Cell lysates were prepared by placing cells in lysis buffer containing 25% Triton X-100 with shaking for 1 h. Lysates were collected and centrifuged at 17,000 rpm for 5 min at 4 °C to pellet cell debris and then added into HEGA buffer for High-Performance Liquid Chromatography (HPLC) analysis.

#### Measurement of catecholamines and metabolites by HPLC

Assay samples were filtered (0.20 μm filter; ThermoFisher Scientific) and analyzed via HPLC with electrochemical detection as previously reported^7^. Briefly, samples were separated on a C18 reverse-phase column (Hypersil ODS C18 column, ThermoFisher Scientific) with MD-TM mobile phase (ThermoFisher Scientific). Catecholamines DA and NE, precursor L-DOPA as well as catecholamine metabolites DOPAC and HVA were detected on a ThermoScientific Dionex UltiMate 3000 ECD-3000RS Electrochemical Detector (ThermoFisher Scientific) at 300 mV oxidation potential. The Chromeleon Chromatography Data System software package (ThermoFisher Scientific, version 7) quantified catecholamine (DA, NE), L-DOPA and metabolite (DOPAC, HVA) content present in each sample from the respective areas under the HPLC peaks based on defined calibration curves.

### Hormone secretion assays and measurement

#### Islet secretion assay

Measurements of hormone secretion were performed using static incubation of pancreatic islets. Following islet isolation and recovery, human or mouse islets were transitioned from complete RPMI 1640 medium (containing 11 mM glucose) to complete RPMI 1640 media supplemented with 25 mM glucose. The increased glucose (25 mM) was used to inhibit α-cell glucagon secretion to diminish glucagon levels to basal levels to improve the dynamic range of the assay and thus effectively unmask the role of ligand-mediated glucagon secretion as described earlier^45^. Islets were then transferred to a 24-well plate containing KRB buffer also supplemented with 25 mM glucose. Drug treatments were added in KRB (25 mM glucose) (1 hr, 37 °C, 5% CO_2_). After treatment, KRB was supplemented with a protease inhibitor cocktail (1 tablet/10 mL KRB; Roche Diagnostics, Mannheim, Germany) to prevent glucagon degradation. Supernatants were collected and placed on ice to further prevent glucagon degradation and precipitation. Undiluted supernatants and 1:2 dilutions were used for the glucagon detection assay, while 1:10 dilutions were used for insulin measurement; all dilutions were in KRB.

#### Glucose-stimulated insulin secretion (GSIS) in INS-1E cells

Insulin secretion assays in INS-1E cells were conducted as described earlier^7,42^. Briefly, cells were seeded into 24-well plates (pre-coated with poly-l-Lysine) in RPMI 1640 complete media at 5 × 10^5^ cells/well and cells were cultured overnight (37˚C, 5% CO_2_). Insulin secretion was carried out 48 h after seeding the cells. On an experimental day, cells were first glucose-starved in KRB (0 mM glucose) for 1 h (37 °C, 5% CO_2_), followed by glucose stimulation with KRB supplemented with 20 mM glucose in the presence or absence of drugs for 90 min (37 °C, 5% CO_2_). The supernatants were collected and diluted 1:10 in KRB for the insulin detection assay.

#### Hormone detection assays

For glucagon detection, we used a commercially available glucagon detection kit (Cisbio Bioassays, Bedford, MA). The assay is based on homogeneous time-resolved fluorescence resonance energy transfer (HTRF) technology. Standard curve and assay samples (10 μL/well) were plated into a 384-well white, low-volume, round-bottom plate (Corning, Corning, NY). The two anti-glucagon antibodies were mixed in a 1:1 donor (cryptate)/acceptor (d2) ratio in the glucagon assay detection buffer (Cisbio Bioassays) and 10 µL of this glucagon antibody mix was added to each well. Secreted insulin was similarly detected and measured by an HTRF approach using the insulin high range detection kit (Cisbio Bioassays) as described previously^42^. The two anti-insulin antibodies were mixed in a 1:2 donor (cryptate)/acceptor (XL665) ratio in the insulin assay detection buffer (Cisbio Bioassays) and 15 µL of the insulin antibody mix was added per well. For both glucagon and insulin HTRF assays, the incubation time was 2 h at room temperature. Plates were read using a PheraStar FSX equipped with an HTRF optic module (337 665 620 mm) (BMG Labtech, Ortenberg, Germany). Integration start was set at 60 microseconds and the integration time was 400 microseconds with 200 flashes/well. The insulin and glucagon concentrations of the assay wells were derived via extrapolation of ratiometric fluorescence readings (665 nm/620 nm) to a second-order quadratic polynomial curve. The raw data were obtained in ng/mL insulin secreted and pg/mL glucagon secreted. Dose-response curves were fit via non-linear regression of Log[ligand] versus normalized % glucagon or insulin secretion. Investigators were blinded to the identity of samples during the initial analysis.

### Quantitative real-time PCR

Total RNA from INS-1E cells and the INS-1E-derived α_2A_-adrenergic receptor knockout cell line were isolated using the RNeasy Plus micro Kit (Qiagen, Valencia, CA). The isolated mRNA was reverse transcribed via the Superscript III First-Strand Synthesis System (ThermoFisher Scientific) according to manufacturers’ instructions. For quantitative real-time PCR (qPCR) assays, expression levels of rat α_2A_-adrenergic receptor (*Adra2a*) were detected using the QuantiTect SYBR Green PCR Kit (Qiagen) and SYBR Select Master Mix (Sigma-Aldrich) and quantified according to the 2^ΔΔCt^ method. For these reactions, we used 0.5 μM primers (*Adra2a* forward primer: 5′-AGCATCGGAAAGACGAACCG-3′ and *Adra2a* reverse primer: 5′-GTGCAAAAGAGCACGTCGAG-3′). PCR products were confirmed in 1.5% agarose gels. Analysis of melting curves confirmed primer specificity. Each assay was run in triplicate and independently repeated ≥3 times to verify the results. Data were normalized to the expression of the commonly used reference gene *Tbp* which encodes TATA-binding protein.

### Radioligand binding assays

Radioligand binding and competition assays were conducted as previously described^46^. Briefly, adherent INS-1E cells expressing the endogenous α_2A_-adrenergic receptors or transfected HEK-293 cells overexpressing human α_2A_-adrenergic receptor were dissociated from plates, and intact cells collected by centrifugation (600–900 g, 5–10 min, 4 °C). Cells were resuspended and lysed using 5 mM Tris-HCl and 5 mM MgCl_2_ (pH 7.4, 4 °C). Cell lysates were pelleted by centrifugation at 30,000 × *g* for 30 min and resuspended in Earle’s Balanced Salt Solution with Ca^2+^ (pH 7.4). For saturation binding assays, cell lysates (100 µL, 2–5 μg of protein for HEK-293 cells and 10–20 μg of protein for INS-1E cells, quantified by the Bradford Assay) were incubated with the indicated concentrations of [^3^H]RX821002 (90 min, room temperature). For competition binding assays, cell lysates were incubated with the indicated concentrations of DA, NE, yohimbine, or clonidine and 0.4–0.9 nM [^3^H]RX821002 (90 min, room temperature). Nonspecific binding was determined in the presence of 10 μM yohimbine. The bound ligand was separated from free ligand by filtration through a Perkin Elmer Unifilter-96 GF/C 96-well microplate using the Perkin Elmer Unifilter-96 Harvester (Perkin Elmer, Waltham, MA), followed by three washes with ice-cold assay buffer. After drying, a liquid scintillation cocktail (MicroScint PS; Perkin Elmer) was added to each well, and plates were sealed and analyzed on a Topcount NXT liquid scintillation counter (Perkin Elmer). *K*_i_ values were calculated from observed IC_50_ values using the Cheng–Prusoff equation^47^.

### NanoBRET

#### DNA constructs

For nanoBRET experiments, we used receptor constructs consisting of either human D2R (*DRD2*) and rat α_2A_-adrenergic receptor (*Adra2a*) cDNAs fused to HaloTag at the C-terminus (α_2A_-AR-HaloTag and D2R-HaloTag). Both receptors were also tagged at the N terminus with an IL6 signal sequence followed by a HiBiT tag. For G protein and β-arrestin2 receptor recruitment studies, we used human Gα_i1_ with nanoluciferase (NanoLuc) inserted at position 91 (NanoLuc-Gα_i1(91)_) and human β-arrestin2 fused with NanoLuc at the N terminus (NanoLuc-β-arrestin2). All constructs were cloned into a pcDNA3.1(+) vector backbone (ThermoFisher Scientific) and confirmed by sequencing analysis.

#### Transfection

HEK-293T cells were cultured in 100-mm dishes and transfected upon 70% confluency. A constant amount of plasmid cDNA (2.5 μg) was transfected into the HEK-293T cells using Lipofectamine 3000 (ThermoFisher Scientific) according to the manufacturer’s instructions. Our nanoBRET assays were performed to detect ligand-induced recruitment of Gα_i_ or β-arrestin2 to either α_2A_-adrenergic receptor or D2R. Cells were co-transfected with the following optimized donor:acceptor nanoBRET pair ratios: 50 (α_2A_-AR-HaloTag): 1 (NanoLuc-Gα_i1(91)_ or NanoLuc-β-arrestin2); and 100 (D2R-HaloTag): 1 (NanoLuc-Gα_i1(91)_ or NanoLuc-β-arrestin2). For NanoLuc-only controls, empty pcDNA3.1(+) vector was used to maintain a constant amount of total transfected DNA.

#### NanoBRET

Cells were harvested, washed, and resuspended in fresh medium 24 h post-transfection. Approximately 5 × 10^4^ cells/well were distributed in pre-coated 96-well plates and allowed to adhere overnight (37 °C, 5% CO_2_). On an experimental day, cells were washed with Hanks’ Balanced Salt Solution (HBSS, Gibco/ThermoFisher Scientific) and the respective receptors were labeled with 100 nM HaloTag NanoBRET 618 ligand (Promega Corporation, Fitchburg, WI) in phenol red-free Opti-MEM I reduced serum medium (Gibco/ThermoFisher Scientific) (2 h, 37 °C, 5% CO_2_). Following labeling, cells were washed once with HBSS, and 5 μM furimazine (substrate for NanoLuc) was added to every well. Drugs were then added to the samples and incubated for 12 min. Plates were read 5 min following drug addition using a PheraStar FSX equipped with a nanoBRET-compatible optic module (LUM 610 450) (BMG Labtech). The nanoBRET ratio was calculated as the emission of the acceptor (618 nm) divided by the emission of the donor (460 nm). The nanoBRET signal from assay wells was corrected by subtracting the 618 nm/460 nm ratio of cells co-expressing NanoLuc and HaloTag minus the nanoBRET ratio of cells expressing only the NanoLuc in the same experiment. The nanoBRET data were normalized to the % maximum response of either NE for the α_2A_-adrenergic receptor experiments or DA for the D2R experiments. NanoBRET data were further normalized to define the minimum and maximum response to the corresponding endogenous ligand, using the GraphPad Prism software package (version 7.02, GraphPad Software, San Diego, CA). EC_50_ values were calculated via a non-linear regression analysis via GraphPad software.

### RNA-sequencing analyses

Transcriptome analyses of human α- and β-cells were conducted using an RNA-sequencing data set that we recently established from dissociated and sorted human α- and β-cells isolated from non-diabetic human pancreata:^48,49^ human α-cells (GEO: GSE106148)^48^; human β-cells (GEO: GSE116559)^49^. Mouse RNA-sequencing data was derived from an available data set: mouse α- and β-cells (GEO: GSE80673)^50^. To calculate the relative expression ratios of genes of interest within each cell type and species, we used the original gene counts from these RNA-sequencing data sets. Counts for genes of interest were normalized to derive expression values while accounting for potential differences in library size or read length bias. Ratios of genes of interest to *Drd2* or *Drd3* were calculated for α- and β-cells from human and mouse using the control samples within each cell type. The difference in ratios relative to *Drd2* or *Drd3* were calculated using the limma software package for each cell type for human and mouse, as described earlier^51^.

### Statistical analyses

GraphPad Prism (version 7.02) was used for all statistical analyses. Two-tailed *t*-tests were used to analyze HPLC results between L-DOPA-treated and untreated samples as well as between secreted and intracellular assay samples. Ordinary one-way ANOVA followed by Dunnett’s multiple comparisons tests were used to analyze differences between the effects of drug treatments on glucagon or insulin secretion from islets. The variance was similar between the groups being statistically compared. Sample sizes were initially chosen on the basis of power analyses assuming an effect size of 0.60, power level of 0.80, and a probability level for statistical significance of 0.05.

## Results

### Human and mouse pancreatic α- and β-cells express the catecholamine biosynthetic and catabolic machinery

**Fig. 1:**
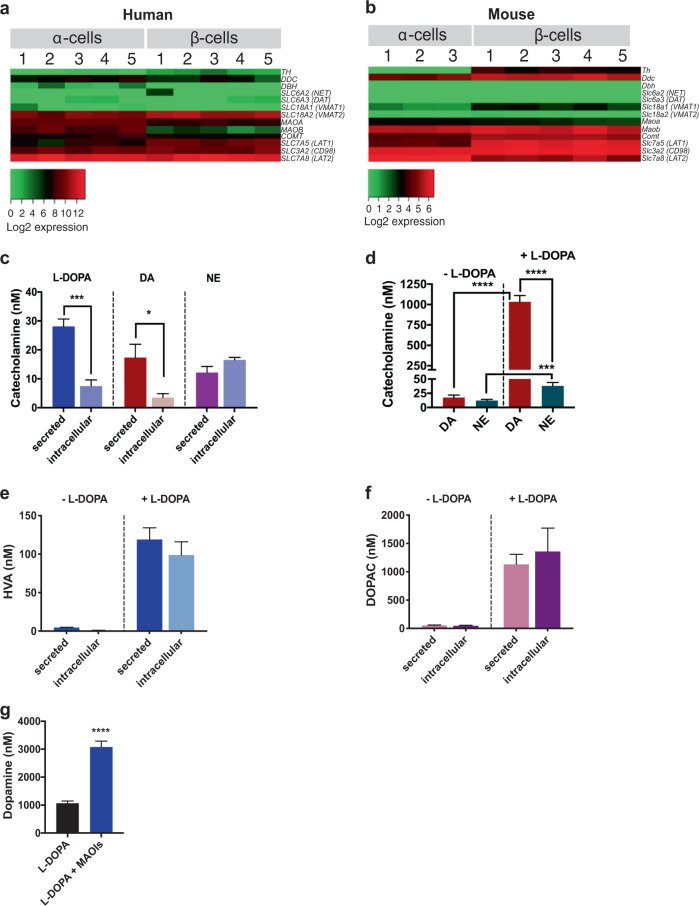
Human and mouse pancreatic α- and β-cells express the catecholaminergic machinery with α-cell production of catecholamines and metabolites. **a**, **b** Transcriptome by RNA-sequencing analysis of purified α- and β-cells from pancreatic islets from **a** non-diabetic human donors (*n* = 5; ages 26–55 years) and **b** mouse. Heatmaps of a selected gene subset focusing on the catecholamine biosynthetic, transport, and vesicular packaging machinery show relative gene expression values in individual α- and β-cell samples of dopaminergic and adrenergic receptors as well as the complete catecholamine biosynthetic and catabolic machinery. **c** HPLC analyses of supernatants and lysates from α-cell-derived αTC1–6 cells demonstrating the synthesis of L-DOPA, DA, and NE de novo in the absence of catecholamine precursor supplementation. Cells secreted most intracellular L-DOPA and DA with significantly lower L-DOPA (*P* = 0.0002) or DA (*P* < 0.05) in lysates compared to supernatants. **d** HPLC analyses show that pre-incubation with 10 μM L-DOPA significantly enhanced α-cell DA and NE production and secretion. Though L-DOPA supplementation boosted NE production (*P* = 0.0004), DA production was preferentially boosted over NE, with DA levels 27-fold more compared to NE (*P* < 0.0001). **e**, **f** In αTC1–6 cells, both secreted and intracellular levels of DA metabolites HVA (**e**) and DOPAC (**f**) were substantially enhanced in response to 10 μM L-DOPA supplementation. **g** Treatment of αTC1–6 cells with a cocktail of monoamine oxidase inhibitors (MAOIs: 10 μM of deprenyl, pargyline, and clorgyline, respectively) significantly enhanced DA synthesis in response to L-DOPA supplementation (blue bar) compared to the 10 μM L-DOPA alone condition (*P* < 0.0001, gray bar). Assay points were carried out in triplicates from *n* ≥ 2 independent experiments. Data are represented as mean ± SEM; two-tailed Student’s *t*-test (**c**, **d**, **g**). *P < 0.05, ***P < 0.001, ****P < 0.0001.

To evaluate whether human and mouse α-cells and β-cells express the machinery of catecholamine biosynthesis and catabolism, we analyzed an RNA-sequencing (RNA-seq) data set that we recently established from human α-cells and β-cells, alongside a comparable available RNA-seq data set from mouse α-cells and β-cells^48–50^. We found both human and mouse α-cells and β-cells express the complete catecholamine signaling machinery, though with major cell type- and species-specific differences (Fig. 1a, b); we confirmed cell-type specificity with enrichment of α-cell- and β-cell-specific markers (e.g., *MAFA, ARX*, *IRX1*, and *IRX2*) in the respective cell types (Supplementary Fig. S1a, b). Both human and mouse α-cells and β-cells express the machinery for catecholamine biosynthesis including tyrosine hydroxylase (*TH*), DOPA decarboxylase (*DDC*), and dopamine β-hydroxylase (*DBH*) (Fig. 1a, b). We also found human and mouse α-cells and β-cells express the L-type amino acid transporter (LAT) isoforms required for cellular uptake of catecholamine precursors (e.g., L-DOPA). This includes α-cell and β-cell expression of both components of the LAT1 heterodimer, LAT1 (*SLC7A5*) and CD98 (*SLC3A2*), as well as LAT2 (*SLC7A8*) (Fig. 1a, b). Similarly, both isoforms of the vesicular monoamine transporters VMAT1 (*SLC18A1*) and VMAT2 (*SLC18A2*) required for vesicular catecholamine uptake are expressed in human and mouse α- and β-cells (Fig. 1a, b). Additionally, in human α-cells and β-cells, we detected the expression of the dopamine transporter (DAT, *SLC6A3*), which enables DA uptake into these cells. In contrast, we found comparatively lower expression of the norepinephrine transporter (NET, *SLC6A2*) in α-cells and β-cells (Fig. 1a, b). Lastly, the catabolic machinery, including monoamine oxidase A and B (*MAOA*, *MAOB*) and catechol-*O*-methyltransferase (*COMT*), is expressed in human and mouse α-cells and β-cells (Fig. 1a, b).

### Pancreatic α-cells synthesize and secrete DA, NE, and L-DOPA

We examined whether α-cells produce and secrete DA using αTC1–6 cells, a glucagon-secreting mouse α-cell line^52^. We and others previously showed that rodent β-cells do not synthesize significant L-DOPA or DA de novo, but produce ample quantities of DA in response to uptake of exogenous L-DOPA^6,7,9^. In contrast, we found αTC1–6 cells produce and secrete L-DOPA de novo as well as convert it to DA and NE (Fig. 1c). As in β-cells^7^, exogenous L-DOPA supplementation (10 μM) promotes a substantial 60-fold increase in α-cell DA secretion (*P* < 0.0001). However, NE is increased only 3-fold in response to L-DOPA addition compared to untreated cells (*P* = 0.0004; Fig. 1d). These results suggest α-cells can adjust catecholamine production based on precursor availability and that this mechanism is preferentially geared towards DA synthesis and release. Additionally, metabolites homovanillic acid (HVA) (Fig. 1e) and 3,4 dihydroxyphenylacetic acid (DOPAC) (Fig. 1f) are produced and secreted, indicating α-cell COMT and MAO activity, respectively. We also observed that MAO inhibitors (10 μM of deprenyl, pargyline, and clorgyline) increase secreted DA ~3-fold relative to untreated cells (*P* < 0.0001; Fig. 1g), further confirming MAO activity in α-cells.

### DA differentially modulates glucagon and insulin secretion in mouse and human islets

**Fig. 2:**
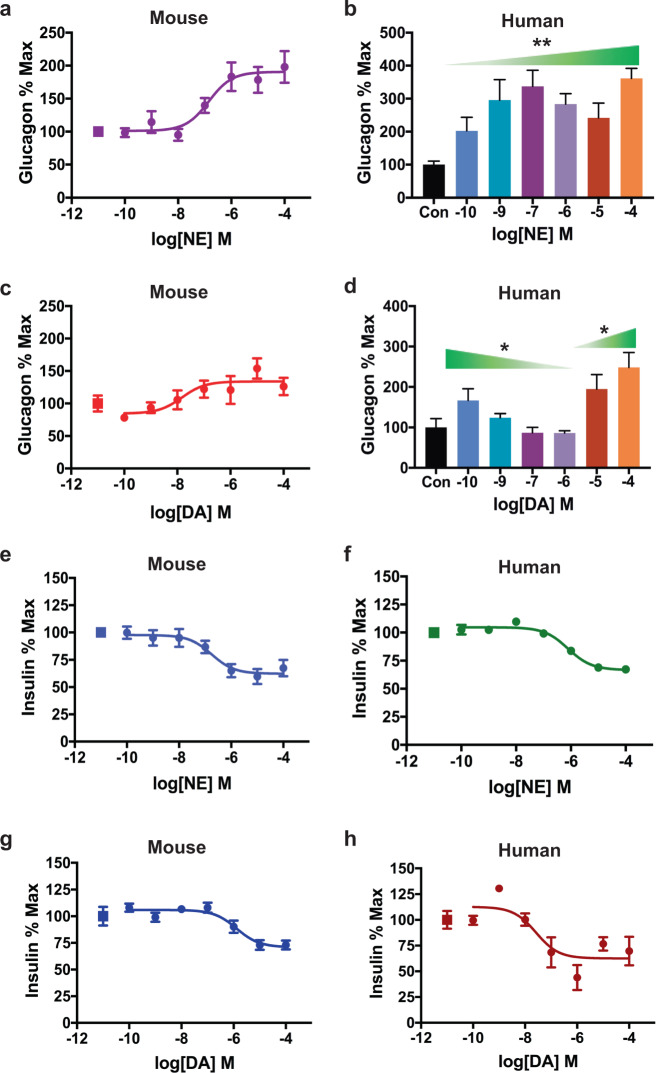
Dopamine and norepinephrine modulate glucagon and insulin secretion in human and mouse islets. **a** Treatment with norepinephrine (NE) produced a dose-dependent increase in secreted glucagon (EC_50_ = 141.7 ± 2.3 nM, *R*^2^ = 0.91) in mouse islets. **b** In human islets, NE treatment also significantly increased α-cell glucagon secretion relative to the vehicle control (Con) [F(6,34) = 4.083, *P* = 0.003]. **c** In mouse islets, treatment with dopamine (DA) dose-dependently enhanced α-cell glucagon secretion (EC_50_ = 14.9 ± 3.8 nM, *R*^2^ = 0.87) in a monophasic manner. **d** In human islets, DA produced a biphasic glucagon response. Low DA concentrations (100 pM–1 μM) progressively diminished α-cell glucagon secretion relative to the vehicle control (Con) [F(4,22) = 3.253; *P* = 0.03]. High DA concentrations (10–100 μM) enhanced glucagon secretion compared to vehicle control [F(2,12) = 5.448; *P* = 0.02]. **e**, **f** NE reduced glucose-stimulated insulin secretion (GSIS) from β-cells in a concentration-dependent manner in: **e** mouse islets (IC_50_ = 178.5 ± 2.4 nM, *R*^2^ = 0.91), and **f** human islets (IC_50_ = 787.2 ± 1.3 nM, *R*^2^ = 0.86). **g**, **h** DA reduced GSIS in **g** mouse islets (IC_50_ = 1.29 ± 0.002 μM, *R*^2^ = 0.83) and **h** in human islets (IC_50_ = 26.2 ± 2.9 nM, *R*^2^ = 0.89). All secretion assays were performed in triplicate from *n* ≥ 3 independent experiments. Representative experiments are shown for all human islet hormone secretion experiments. Glucagon data normalized to % maximal secreted glucagon; insulin data normalized to % maximal secreted insulin. In **a**, **c**, **e**–**h** squares represent vehicle-treated controls. Data are represented as means for all experimental replicates ± SEM; one-way ANOVA followed by Dunnett’s multiple comparisons test (**b**, **d**). *P < 0.05, **P < 0.01.

Since α-cells produce DA and NE, we examined whether these catecholamines regulate glucagon secretion. We found NE produces a dose-dependent stimulation of glucagon secretion in mouse islets (EC_50_ = 142 ± 2.3 nM; Fig. 2a), consistent with earlier studies^53,54^. Similarly, increasing NE concentrations significantly enhance glucagon secretion in human islets from non-diabetic donors (Supplementary Table S1 and Fig. 2b).

We found that DA also modulates α-cell glucagon secretion, though with important species-specific differences. Whereas DA enhances glucagon release from mouse islets in a monophasic manner (EC_50_ = 14.9 ± 3.8 nM; Fig. 2c), we observed a biphasic glucagon secretory response to DA in human islets (Fig. 2d): low DA concentrations (100 pM–1 μM) reduce glucagon secretion (*P* = 0.03), while higher DA concentrations (10–100 μM) enhance glucagon release (*P* = 0.03) (Fig. 2d). We examined insulin secretion from the same mouse and human islets, finding that NE reduces insulin secretion in a monophasic manner in both mice (IC_50_ = 179 ± 2.4 nM; Fig. 2e) and human islets (IC_50_ = 787 ± 1.3 nM; Fig. 2f). DA also dose-dependently inhibits insulin secretion in mouse islets, though with ~7-fold lower potency compared to NE (IC_50_ = 1.3 ± 0.002 μM; Fig. 2g). Additionally, we found that DA inhibits insulin release in human islets (IC_50_ = 26.2 ± 2.9 nM; Fig. 2h) and that this inhibition is monophasic, unlike the DA’s biphasic glucagon response.

### Differences in DA and NE receptor expression in mouse and human islet α- and β-cells

Recent evidence suggests that DA can signal not only through D_2_-like receptors, but also via adrenergic receptors, albeit with different potencies^46^. Importantly, both dopaminergic and adrenergic receptor subtypes are co-expressed in human and rodent α-cells and β-cells^5,7,9,22,23^. Therefore, we compared cell type- and species-specific expression of α- and β-adrenergic receptor subtypes relative to either D2R or D3R using our human and mouse α-cell and β-cell RNA-seq data sets. We found substantial differences in the expression of D_2_-like and adrenergic receptors in mouse versus human α-cells and β-cells (Supplementary Fig. S2). β_1_-adrenergic receptor and D2R are expressed at similar levels in human α-cells (e.g., β_1_-adrenergic receptor/D2R ratio = 1.25; Supplementary Fig. S2a), while there is 74-fold higher expression of β_1_-adrenergic receptor relative to D2R in mouse α-cells (Supplementary Fig. S2b). Likewise, in human β-cells, α_2A_-adrenergic receptor is only 1.5-fold more expressed than D2R (Supplementary Fig. S2a) but, in mouse β-cells, α_2A_-adrenergic receptor is 1,395-fold more expressed compared to D2R (Supplementary Fig. S2b). We found similar expression ratios comparing adrenergic receptors relative to D3R in mouse versus human α- and β-cells (Supplementary Fig. S2c, d).

Based on these relative differences in receptor expression, we hypothesized that in human α-cells, DA promotes a biphasic glucagon response by activating: (1) inhibitory D_2_-like receptors that readily bind DA at lower concentrations, which leads to secretory inhibition; and (2) stimulatory β-adrenergic receptors that are more effectively activated by DA at higher concentrations, resulting in enhanced glucagon release. In contrast, since mouse α-cells mainly express stimulatory β_1_-adrenergic receptors, DA primarily stimulates glucagon secretion. Thus, the predominantly expressed β_1_-adrenergic receptors will mask any potential inhibition from DA’s activation of D_2_-like receptors, which are expressed at much lower levels. This would explain the observed monophasic enhancement of glucagon release (Fig. 2c). Dopaminergic stimulation of adrenergic receptors also extends to β-cells. Because mouse β-cells express far more inhibitory α_2A_-adrenergic receptors relative to D2R or D3R (Supplementary Fig. S2), our data suggest that DA primarily acts on mouse α_2A_-adrenergic receptors which also diminish insulin release. On the other hand, human β-cells express considerably more D_2_-like receptors compared to mouse β-cells; levels of D_2_-like receptors and α_2A_-adrenergic receptors are similar in human β-cells. Since human β-cell D_2_-like receptors can be activated by DA at presumably lower concentrations than α_2A_-adrenergic receptors, this may account for DA’s 50-fold higher potency for decreasing insulin secretion in human islets compared to mouse (Fig. 2g, h).

### DA acts via adrenergic receptors to regulate hormone secretion in pancreatic islets

**Fig. 3:**
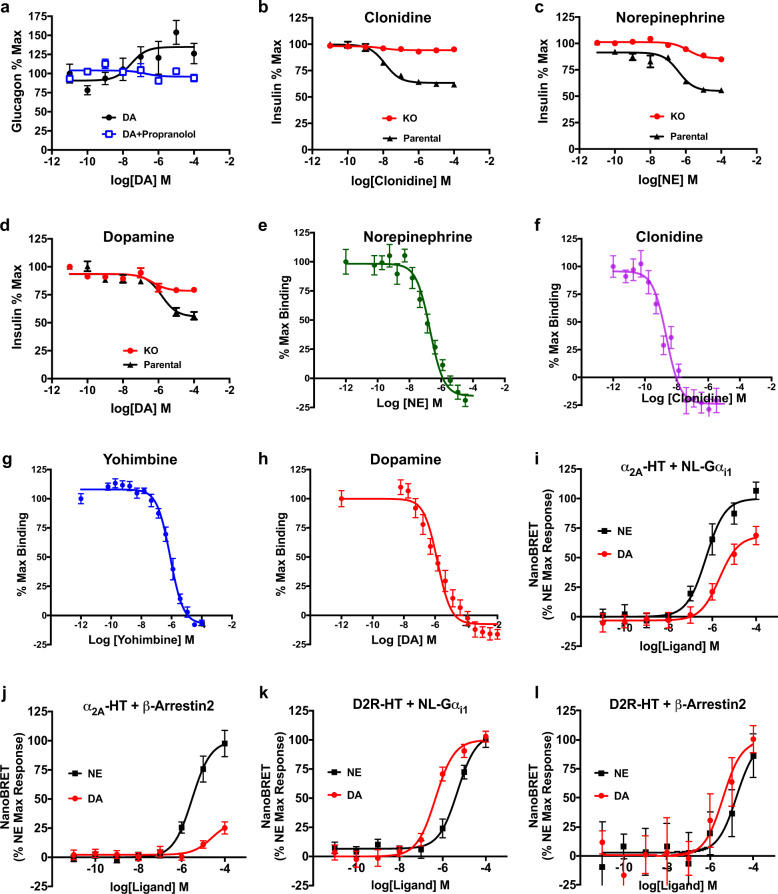
Dopamine signals through the adrenergic system to modulate insulin and glucagon secretion. **a** β-adrenergic receptor antagonist propranolol (100 nM) eliminated DA-induced increases in α-cell glucagon secretion from mouse islets (in blue) compared to treatment with DA alone (in black; EC_50_ = 14.9 ± 3.8 nM, *R*^2^ = 0.68). Glucagon data were normalized to % maximal secreted glucagon. **b** CRISPR-Cas9-mediated knockout (KO) of endogenously expressed α_2A_-adrenergic receptors in insulin-secreting INS-1E cells (in red) attenuated clonidine’s ability to diminish glucose-stimulated insulin secretion (GSIS) compared to the parental INS-1E cells (in black; IC_50_ = 12.7 ± 1.3 nM, *R*^2^ = 0.92). **c** Efficacy of GSIS inhibition by norepinephrine (NE) was diminished 2.9-fold along with decreased potency in the α_2A_-adrenergic receptor KO cells (in red; IC_50_ = 1.4 ± 0.002 nM, *R*^2^ = 0.70) compared to the parental INS-1E cells (in black; IC_50_ = 39.8 ± 1.5 nM, *R*^2^ = 0.90). **d** Efficacy of dopamine (DA)-induced GSIS inhibition was reduced 2.4-fold, while potency was increased in the α_2A_-adrenergic receptor KO cells (in red; IC_50_ = 474 ± 2.5 nM, *R*^2^ = 0.75) compared to the parental INS-1E cells (in black; IC_50_ = 1.5 ± 0.002 μM, *R*^2^ = 0.89). For **b**–**d**, insulin data was normalized to % maximal secreted insulin. **e**–**h** Radioligand binding of adrenergic and dopaminergic ligands to endogenous α_2_-adrenergic receptors in INS-1E cells. Competition curves of α_2A_-adrenergic receptor [^3^H]RX821002 versus increasing concentrations of free competitors: **e** NE (*K*_i_ = 22.5 ± 1.2 nM); **f** clonidine (*K*_i_ = 0.27 ± 0.001 nM); **g** yohimbine (*K*_i_ = 92.2 ± 1.1 nM); **h** DA (*K*_i_ = 164 ± 1.2 nM). Radioligand experiments were normalized to % maximal binding with all assays performed in triplicate in *n* ≥ 3 independent experiments. Error bars = SEM. **i**–**l** Concentration-response nanoBRET assays examining ligand-stimulated G protein and β-arrestin2 receptor recruitment in HEK-293T cells transiently transfected with either HaloTag-labeled α_2A_-adrenergic receptor (α_2A_-HT) or D2R (D2R-HT) and NanoLuc-labeled Gα_i1_ (NL-Gα_i1_) versus β-arrestin2 (NL-β-arrestin2) as the respective nanoBRET pairs. **i** DA treatment caused dose-dependent Gα_i1_ recruitment to α_2A_-adrenergic receptor, albeit with reduced potency and efficacy compared to NE (DA: in red, EC_50_ = 2.1 ± 0.002 μM, *R*^2^ = 0.77; NE: in black, EC_50_ = 520 ± 1.4 nM, *E*_max_ = 71.5%, *R*^2^ = 0.82). **j** NE treatment produced dose-dependent increases in β-arrestin2 recruitment to α_2A_-adrenergic receptor (in black, EC_50_ = 3.1 ± 0.001 μM, *R*^2^ = 0.72), while DA produced a negligible response (in red). **k** DA and NE treatments both resulted in comparable Gα_i1_ recruitment to D2R, with DA more potent (DA in red; EC_50_ = 471 ± 1.3 nM, *R*^2^ = 0.82) than NE (NE in black; EC_50_ = 4.9 ± 0.001 μM, *R*^2^ = 0.67). **l** Both DA and NE treatments stimulated β-arrestin2 recruitment to D2R with DA more potent (DA in red; EC_50_ = 3.9 ± 0.003 μM, *R*^2^ = 0.67) compared to NE (NE in black; EC_50_ = 16.8 ± 0.004 μM, *R*^2^ = 0.63). NanoBRET data were baseline-corrected by subtracting the nanoBRET ratio from the NanoLuc-only wells from the ratio calculated from assay wells expressing both NanoLuc and HaloTag. Results for α_2A_-adrenergic receptor recruitment were normalized to % maximal NE response; data for D2R recruitment were normalized to % maximal DA response. Data are represented as means ± SEM for all experimental replicates and were performed in triplicate from *n* ≥ 3 independent experiments.

We functionally tested DA’s ability to directly signal through islet adrenergic receptors to modulate hormone secretion. Since the β_1_-adrenergic receptor is the predominant catecholamine receptor subtype expressed in mouse α-cells, we pre-treated mouse islets with the β-adrenergic antagonist propranolol (100 nM) prior to DA treatment. We found that propranolol attenuates DA’s enhancement of glucagon secretion (Fig. 3a). This suggests that DA mainly acts on β_1_-adrenergic receptors in mouse α-cells to modulate glucagon secretion, potentially masking contributions from the less abundant DA receptors also expressed in these cells.

To dissect DA signaling via dopaminergic versus adrenergic receptors in β-cells, we deleted the α_2A_-adrenergic receptor via CRISPR-Cas9 in rodent β-cell-derived INS-1E cells. We generated a series of clonal lines and selected a complete α_2A_-adrenergic receptor knockout (KO) line based on the total loss of receptor mRNA expression by qPCR (Supplementary Fig. S3a). We also functionally validated this α_2A_-adrenergic receptor KO cell line by testing the effects of clonidine, a selective α-adrenergic receptor agonist, on GSIS. While clonidine potently reduces GSIS in the parental INS-1E cell line (IC_50_ = 12.7 ± 1.3 nM), the drug’s GSIS inhibition is virtually eliminated in the α_2A_-adrenergic receptor KO cells (Fig. 3b and Supplementary Table S2). Clonidine’s small residual effects on GSIS in the KO cells are likely due to the drug’s actions on remaining α_2_-adrenergic receptors including α_2C_-adrenergic receptors which play a small role in inhibiting GSIS in β-cells^55,56^. Like clonidine, NE also dose-dependently reduces GSIS in the parental INS-1E cells (IC_50_ = 39.8 ± 1.5 nM; Fig. 3c). Unlike clonidine, though NE’s efficacy and potency are significantly diminished (35-fold decrease in efficacy, IC_50_ = 1.4 ± 0.002 μM), NE’s GSIS inhibition is not entirely eliminated in the KO cells (Fig. 3c). This suggests that NE acts at additional catecholamine receptors, albeit with lower potency. DA’s efficacy in inhibiting GSIS inhibition is also reduced (2.4-fold) but not eliminated in the KO cells compared to the parental line (Fig. 3d). DA’s potency is greatly increased in the KO cells (IC_50_ = 474 ± 2.5 nM) compared to the parental INS-1E cells (IC_50_ = 1.5 ± 0.002 μM). Together, these data suggest that much of DA’s efficacy in reducing GSIS is attributable to its actions on α_2A_-adrenergic receptors in β-cells. Importantly, the deletion of the α_2A_-adrenergic receptor in the KO cells unmasks the higher potency of DA for D2R, also expressed in INS-1E cells^5^, and reveals the relative contribution of D2R in the regulation of GSIS (Supplementary Table S2).

### DA binds to endogenous β-cell α_2A_-adrenergic receptors

We conducted radioligand binding studies employing the α_2_-adrenergic receptor antagonist [^3^H]RX821002 to determine the binding affinity of DA at endogenous β-cell α_2A_-adrenergic receptors. We first confirmed the binding of [^3^H]RX821002 at α_2A_-adrenergic receptors using membranes from HEK-293 cells overexpressing α_2A_-adrenergic receptors and determined the binding affinity (*K*_D_ = 0.67 ± 0.05 nM) and B_max_ (5691 ± 103 fmol/mg protein) values (Supplementary Fig. S3b). Competition experiments of [^3^H]RX821002 with α_2_-adrenergic receptor antagonist yohimbine and DA, in the same α_2A_-adrenergic receptor-overexpressing HEK-293 cells, demonstrated that both yohimbine and DA displace [^3^H]RX821002 with differing affinities: nanomolar affinity for yohimbine (*K*_i_ = 38.2 ± 1.1 nM) and micromolar affinity for DA (*K*_i_ = 22.1 ± 0.001 μM) (Supplementary Fig. S3c). This suggests that DA targets α_2A_-adrenergic receptors as a lower-affinity substrate compared to a classical α_2_-adrenergic receptor-ligand like yohimbine. We also compared the binding of [^3^H]RX821002 at endogenous α_2A_-adrenergic receptors expressed in the parental INS-1E cells versus the α_2A_-adrenergic receptor KO cells. Our studies confirm substantially diminished radioligand binding in membranes from the KO cells (*B*_max_ = 29.1 ± 4.3 fmol/mg protein) relative to the parental INS-1E cells (*B*_max_ = 110 ± 3.3 fmol/mg protein) (Supplementary Fig. S3d). We next determined binding affinities of several α_2_-adrenergic receptor ligands (NE, clonidine, yohimbine) to endogenous α_2A_-adrenergic receptors in INS-1E cells using the calculated *K*_D_ from the saturation binding experiments in these cells (*K*_D_ = 0.10 ± 0.02 nM) (Supplementary Fig. S3d and Supplementary Table S3). NE (Fig. 3e), clonidine (Fig. 3f), and yohimbine (Fig. 3g) all displace [^3^H]RX821002 in competition experiments using INS-1E cell membranes (NE: *K*_i_ = 22.5 ± 1.2 nM; Clonidine: *K*_i_ = 0.27 ± 0.001 nM; yohimbine: *K*_i_ = 92.2 ± 1.1 nM). Significantly, DA also successfully displaces [^3^H]RX821002 from endogenous receptors (*K*_i_ = 164 ± 1.2 nM; Fig. 3h), validating DA’s ability to bind endogenously expressed α_2A_-adrenergic receptor. Our data demonstrate an order of affinity of clonidine > NE > yohimbine > DA and suggest that α_2A_-adrenergic receptors can serve as both low-affinity targets for DA and high-affinity targets for classical α_2_-adrenergic ligands like NE, clonidine, and yohimbine.

### DA is a Gα_i_-biased ligand at α_2A_-adrenergic receptors

We determined whether DA and NE differ in their abilities to initiate intracellular signaling upon activation of D2R and α_2A_-adrenergic receptors. Using nanoBRET technology, we focused on ligand-stimulated receptor recruitment of Gα_i_ and β-arrestin2, initiators of G protein-dependent and G protein-independent signaling pathways, respectively^56^. α_2A_-adrenergic receptors were labeled with HaloTag, and either Gα_i1_ or β-arrestin2 were labeled with the highly-sensitive nanoluciferase (NanoLuc) as the nanoBRET pair. We found that α_2A_-adrenergic receptor activation by DA or NE results in Gα_i1_ recruitment to the receptor, with NE showing higher potency (EC_50_ = 520 ± 1.4 nM) and efficacy compared to DA (EC_50_ = 2.1 ± 0.002 μM; Fig. 3i). Significantly, while NE stimulation causes receptor recruitment of β-arrestin2 (EC_50_ = 3.1 ± 0.001 μM), DA stimulation does not (Fig. 3j). To exclude potential kinetic differences in the ability of DA to recruit β-arrestin2 versus Gα_i1_, time-course experiments showed that receptor stimulation by DA does not result in the recruitment of β-arrestin2 at any of the time points tested (data not shown). By comparison, stimulation of D2R by either DA or NE leads to recruitment of both Gα_i1_ and β-arrestin2, with DA showing higher potency than NE (Gα_i1_: DA EC_50_ = 471 ± 1.3 nM, NE EC_50_ = 4.9 ± 0.001 μM; β-arrestin2: EC_50_ = 3.9 ± 0.003 μM, NE EC_50_ = 16.8 ± 0.004 μM) (Fig. 3k, l). Overall, our results suggest that DA functions uniquely at α_2A_-adrenergic receptors as a G protein-biased agonist by selectively directing intracellular signaling towards G protein-mediated transduction pathways.

### APDs increase α-cell glucagon and β-cell insulin secretion in human islets

**Fig. 4:**
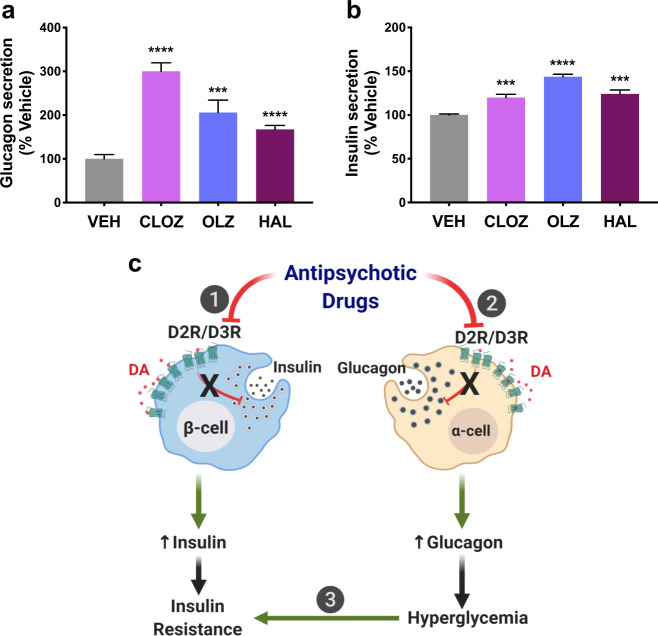
Antipsychotic drugs increase pancreatic insulin and glucagon secretion in human islets. **a** Antipsychotic drugs (APDs) clozapine (CLZ), olanzapine (OLZ), and haloperidol (HAL) (all 1 µM) significantly raised α-cell glucagon secretion in isolated human islets (CLZ: *P* < 0.0001; OLZ: *P* = 0.0002; HAL: *P* < 0.0001); and **b** raised glucose-stimulated insulin secretion from β-cells relative to vehicle controls, in the same human islets (CLZ: *P* = 0.0006; OLZ: *P* < 0.0001; HAL: *P* = 0.0004). Results are normalized to the vehicle. Data are represented as means ± SEM; two-tailed Student’s *t*-test (**a, b**). ***P < 0.001, ****P < 0.0001. **c** Schematic summarizing APDs’ actions on islet α- and β-cells. (1) In β-cells, APDs block inhibitory D2R/D3R, which ordinarily inhibit insulin release in response to DA stimulation. APDs thus disinhibit insulin release, leading to increased secreted insulin. Over time, this desensitizes insulin-sensitive organs to promote insulin resistance. (2) In parallel, APDs act directly on α-cells, disrupting inhibitory D2R/D3R signaling and elevating glucagon release. (3) The resulting increases in secreted glucagon produce hyperglycemia, which further exacerbates insulin resistance, and leads to an overall worsening of APD-induced dysglycemia.

We determined the effects of D_2_-like receptor antagonism on α-cell glucagon and β-cell insulin secretion in human islets using the APDs haloperidol, olanzapine, and clozapine. All three APDs significantly increase α-cell glucagon release (Fig. 4a). Compared to vehicle, clozapine raises glucagon secretion most (200.2 ± 21.7% increase, *P* < 0.0001), followed by olanzapine (105.7 ± 23.4% increase, *P* = 0.0002) and haloperidol (67.1 ± 13.6% increase, *P* < 0.0001) (Fig. 4a). In parallel, these APDs also significantly increase GSIS from the same human islets compared to the vehicle: clozapine (19.8 ± 4.0% increase, *P* = 0.0006), olanzapine (43.6 ± 3.1% increase, *P* < 0.0001), and haloperidol (24.1 ± 4.6 increase, *P* = 0.0004) (Fig. 4b). Importantly, our findings in human islets are consistent with earlier reports of APD-induced hyperinsulinemia and hyperglucagonemia in both rodent models and in humans^19,21,41,57^. Overall, these results strongly suggest that APDs act directly on both α- and β-cells to disrupt the regulation of glucagon and insulin secretion. In β-cells, APDs block inhibitory D_2_-like receptors, leading to increased insulin secretion. In parallel, APDs disrupt inhibitory α-cell D_2_-like receptor signaling and elevate glucagon release, resulting in hyperglycemia that may further exacerbate insulin resistance (Fig. 4c).

## Discussion

DA signaling in the periphery is increasingly recognized as having critical roles in metabolic regulation^3,4,7,30,33,34,58,59^. Our results offer new insights into outstanding questions concerning the sources of DA and its related catecholamine NE for local α-cell and β-cell adrenergic and dopaminergic signaling in the pancreas. Historically, peripheral catecholamine signaling in the pancreas focused primarily on autonomic innervation of islets^26,27,60^. NE from sympathetic inputs acts on α-cell and β-cell adrenergic receptors to raise glucagon, suppress insulin release, and ultimately elevate blood glucose^25,61^. Sympathetic nervous innervation was therefore long considered to be the key source of NE acting on the endocrine pancreas^25,26^. However, there is increasing awareness that the precise extent of islet innervation is highly variable and species-dependent^26–29,31^. Compared to rodents, recent studies suggest much sparser innervation of human islets^27,30^. Furthermore, most sympathetic axons innervating the pancreas are associated with the smooth muscle cells of local blood vessels rather than local islet cells^26,27,30,62^. Islets also lack direct dopaminergic innervation in either humans or rodents^27,33^. Together, these studies suggest that autonomic sympathetic innervation cannot be the sole source of pancreatic catecholamines. Rather, growing evidence suggests that de novo local production of NE and DA by islet cells is a key source of catecholamines in the endocrine pancreas.

We and others previously showed that rodent and human β-cells express the machinery for DA biosynthesis including TH and AADC and produce DA independently of autonomic inputs^6,7,9,63^. Here, we demonstrate for the first time that human and mouse α-cells also express the catecholamine biosynthetic machinery as well as the LAT1 and LAT2 transporters which are instrumental in cellular L-DOPA uptake^7,64–66^. Functionally, we show that α-cells produce both L-DOPA and DA de novo and significantly boost DA production in response to exogenous L-DOPA supplementation. In contrast, both in vitro and in vivo studies suggest that β-cells synthesize little L-DOPA de novo but instead rely on uptake of precursors from the peripheral circulation to drive DA production^6,7,9,33,34,59^. Remarkably, after meals, blood L-DOPA and DA levels increase >50-fold in humans and rodents^67–71^, allowing β-cells to tune DA synthesis and signaling to meal size^7^. Nevertheless, because α-cells and β-cells exist in close proximity within islets^72,73^, we speculate that α-cells can directly supply their de novo-synthesized L-DOPA and DA to nearby β-cells. Indeed, our data showing that human β-cells express DAT may also be consistent with such a possibility, enabling rapid β-cell uptake of locally-produced islet DA. Moreover, the expression of DAT in α-cells could provide an additional layer of control over local DA signaling by facilitating rapid α-cell DA reuptake to more effectively control exposure to islet DA. Ultimately, our proposed paracrine mechanism would enable rapid, dynamic coordination of intra-islet DA signaling to further fine-tune regulation of insulin and glucagon secretion. Future experiments are needed to test these possibilities.

We also show that mouse α-cells produce NE both de novo and in response to L-DOPA treatment since L-DOPA is a precursor for NE as well as for DA^36,71^. Yet, while L-DOPA uptake substantially boosts α-cell DA production (60-fold), there is considerably less enhancement in NE production (3-fold). This suggests that α-cells preferentially produce DA over NE despite expressing DBH, the enzyme responsible for converting DA to NE. We similarly did not find evidence of significant NE production in β-cells in our earlier studies^7^. Yet, despite the preference for DA production, α-cells and β-cells still express adrenergic receptors, and for some receptor subtypes, to a significantly greater degree relative to D2R/D3R. These data suggest a mismatch between the DA and NE produced by α- and β-cells and the catecholamine receptors expressed by the same cells. Similar mismatches have been described in the brain including regions of the dorsal striatum and prefrontal cortex which express high densities of α_2_-adrenergic receptors despite a paucity of noradrenergic nerve terminals and low levels of extracellular NE; rather, these regions exhibit dense dopaminergic innervation^46,74–76^. It has been postulated that DA serves as the endogenous substrate for these adrenergic receptors^46,77^. Several lines of evidence support this conclusion: (1) In vitro studies demonstrated that DA both binds and signals via α_2_-adrenergic receptors, albeit at lower affinities than NE^46,78–80^. (2) DA has been shown to signal via immune cell and cardiac β-adrenergic receptors^81,82^. (3) Computational modeling studies demonstrate strong structural homologies between DA’s interactions at binding sites of adrenergic receptors and D_2_-like receptors^46,83^.

Our radioligand binding data provides further support for DA’s ability to signal via adrenergic receptors. We demonstrate that DA binds to endogenous β-cell α_2A_-adrenergic receptors in the same range previously reported in mammalian brain^46,84^. Moreover, DA’s actions at β-cell α_2A_-adrenergic receptors are functionally relevant. CRISPR-Cas9 deletion of β-cell α_2A_-adrenergic receptor in INS-1E cells reduces DA’s ability to inhibit GSIS in the KO cells. Nevertheless, despite the loss of α_2A_-adrenergic receptor expression, treatment with DA still causes smaller, though appreciable, dose-dependent inhibition of GSIS, and with higher potency. This suggests that the residual inhibition produced by DA in the α_2A_-adrenergic receptor KO background unmasks the relative contributions of the inhibitory Gα_i_-coupled β-cell DA D_2_-like receptors that remain expressed.

Our nanoBRET findings demonstrate that DA activation of the α_2A_-adrenergic receptor primarily results in recruitment of Gα_i1_ to the receptor with negligible β-arrestin2 recruitment while NE treatment causes robust recruitment of both the G protein and β-arrestin2. These data suggest for the first time that DA functions as a biased ligand when it signals specifically via the α_2A_-adrenergic receptor. Such results are consistent with growing evidence that alternate ligands for GPCRs exhibit biased agonism. Indeed, alternate ligands for the galanin receptor (e.g., spexin) exhibit biased agonism toward G protein signaling compared to galanin^85^. Similar findings have been demonstrated by agouti-related peptide through melanocortin-3 and -4 receptors (MCR3, MCR4) or by endogenous opioid peptides at κ- and μ-opioid receptors^86–88^. Importantly, DA’s signaling bias provides an additional rationale for α-cell and β-cell catecholamine receptor preferences for signaling via DA or NE. Since β-arrestin2 recruitment is important for GPCR desensitization and internalization^89^, we propose that DA’s inability to effectively recruit β-arrestin2 to activated α_2A_-adrenergic receptors may limit receptor desensitization. This can lead to sustained receptor signaling at the cell surface. We, therefore, speculate that these signaling differences between DA and NE may provide islet cells with an additional mechanism for finer temporal control of hormone release that may be especially critical for differential metabolic adaptations to states of acute and chronic stress when concentrations of circulating catecholamines are altered.

**Fig. 5:**
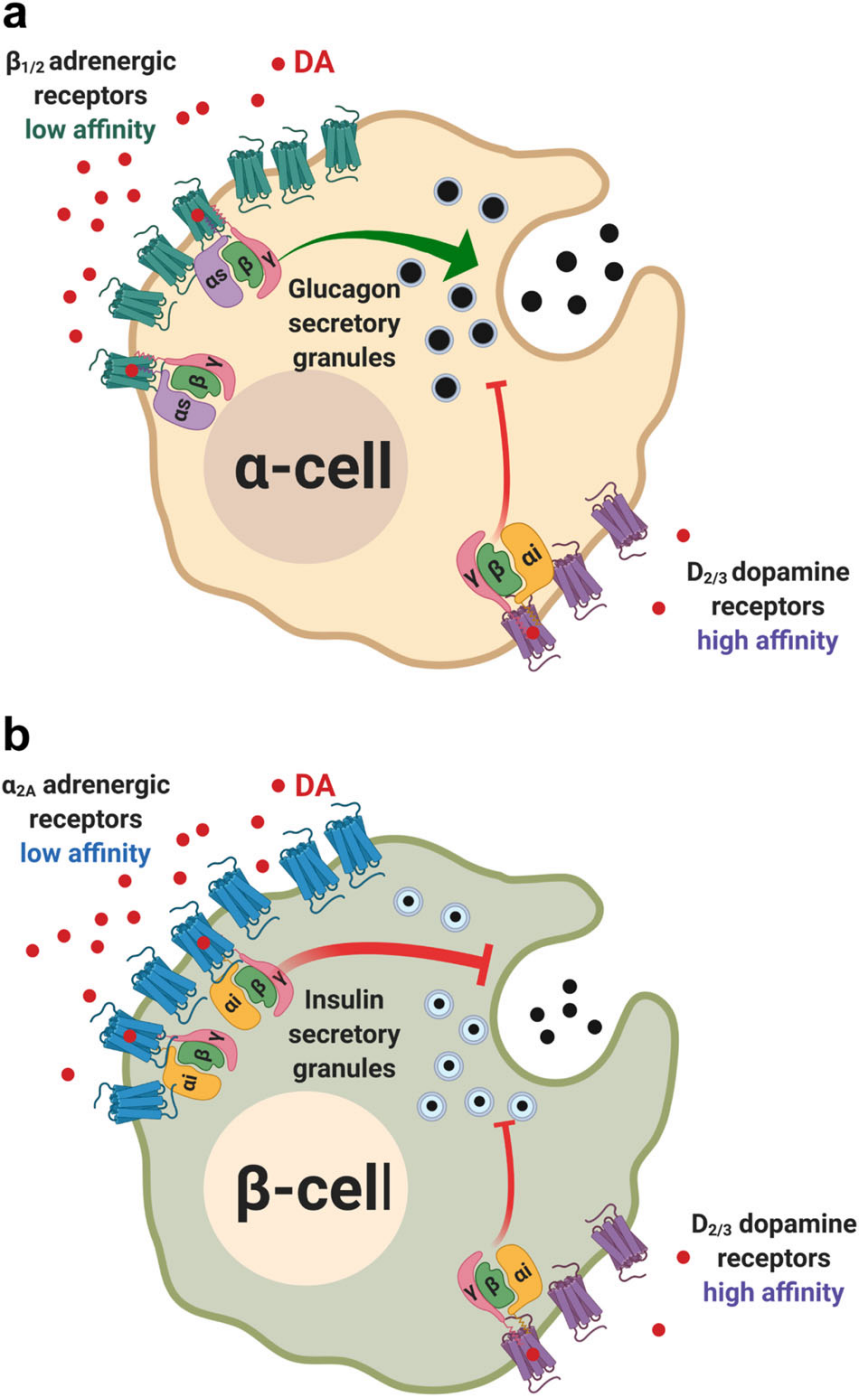
Model for dopamine-mediated regulation of pancreatic α- and β-cell hormone secretion. **a** In α-cells, low concentrations of local dopamine (DA) are sufficient to stimulate high-affinity D_2_-like receptors to diminish glucagon secretion via inhibitory Gα_i_-mediated intracellular signaling. At higher concentrations, there is sufficient DA to trigger the activation of stimulatory β-adrenergic receptors. This results in increased glucagon secretion via stimulatory Gα_s_-mediated signaling. **b** In β-cells, at low DA concentrations, DA primarily signals through the high-affinity D_2_-like receptors to inhibit insulin secretion. At higher DA concentrations, the lower-affinity α_2A_-adrenergic receptors are also activated to maintain overall inhibition of insulin release since both high- and low-affinity catecholamine receptors are coupled to inhibitory Gα_i_.

On the basis of our receptor binding and signaling data, we offer a model of dopaminergic hormone regulation in human islets (Fig. 5). Adequate expression of D2R and D3R in human α-cells enables the high-affinity binding of DA even at low concentrations, leading to inhibitory Gα_i_ signaling that diminishes glucagon release. At higher DA concentrations, there is sufficient DA to occupy β-adrenergic receptors. These α-cell adrenergic receptors effectively function as lower-affinity DA targets, and their activation results in receptor recruitment of stimulatory Gα_s_ that results in enhanced glucagon release. Consequently, the combined actions of DA at α-cell adrenergic and dopaminergic receptors produces a biphasic glucagon response (Fig. 5a). By contrast, in human β-cells, low DA concentrations primarily direct signaling through the high-affinity D2R/D3R to inhibit insulin secretion. However, at higher DA concentrations, DA activates the lower-affinity α_2A_-adrenergic receptors. Because both β-cell D_2_-like and α_2A_-adrenergic receptors are coupled to inhibitory Gα_i_, activation of either receptor type still inhibits insulin secretion (Fig. 5b). In such a model, DA’s ability to selectively signal via high- and low-affinity receptor populations enables α-cells and β-cells to coordinate with one another to finely tune hormone release according to local DA availability.

In addition to α-cells and β-cells, there is increasing evidence suggesting that other pancreatic islet cell types also express D_2_-like receptors. In particular, both human and rodent somatostatin (SST)-secreting δ-cells abundantly express D2R^22,90,91^. Earlier work in rat islets showed that DA inhibits SST secretion via its actions on D_2_-like receptors^92^. This raises the possibility that the DA synthesized by α- and β-cells also locally activates δ-cell D2R to inhibit SST secretion and thus removes SST-mediated inhibition of insulin and glucagon release^32,91,93–98^. Such paracrine DA signaling that involves α-, β-, and δ-cells may offer an additional dimension to our understanding of the intra-islet regulation of hormone secretion. Future studies will further explore such paracrine DA crosstalk between α-, β-, and δ-cells.

Lastly, we demonstrate that APDs clozapine, olanzapine, and haloperidol all significantly elevate secretion of glucagon in human islets. Our findings validate earlier work showing that APD blockade of β-cell D2R/D3R elevates insulin secretion from rodent and human islets^6,7^. Just as importantly, we now find that APDs act directly on an additional target in the periphery, pancreatic α-cells, where D2R/D3R antagonism by these drugs increases glucagon release. Our results provide further evidence of a D2R/D3R-dependent inhibitory mechanism that regulates α-cell hormone secretion, and that disruption of this signaling by APDs increases glucagon release. Indeed, inappropriately elevated blood glucagon levels are a characteristic feature of APD treatment in humans and rodents in vivo^16,19–21^. These APD-induced increases in glucagon occur despite concurrent increases in blood insulin and glucose – conditions that ordinarily decrease glucagon^19,21^. APD-elevated glucagon may therefore drive the hyperglycemic states that these drugs produce since glucagon receptor KO mice are protected against APD-induced hyperglycemia independently of changes in insulin levels^21^. Notably, the APD-dependent increases in human islet glucagon secretion observed here also correlate with the relative metabolic liabilities of these APDs with clozapine > olanzapine > haloperidol^3,4^. Such findings further suggest an important role for drug-induced derangements of α-cell function in producing clinically relevant metabolic disturbances.

Limitations of our work include the possibility that catecholamine signaling in α-cells in vivo differs from the signaling we observed either in ex vivo human or mouse islet preparations or in vitro in tissue culture experiments. Moreover, since we used human islets from cadaveric donor pancreata, there could be potential confounding factors including postmortem interval and cause of death that may impact islet function. Additionally, aside from the APDs used in our present studies, the impacts of APDs including partial D_2_-like receptor agonists (e.g., aripiprazole, brexpiprazole)^99,100^ and other second-generation antipsychotics with a lower risk for weight gain (e.g., ziprasidone)^101^ on α- and β-cell DA signaling have yet to be studied. Future work will therefore examine these outstanding issues.

In summary, the results presented here establish DA as a modulator of glucagon and insulin secretion via its actions on both adrenergic and dopaminergic receptors in human and mouse islets. Furthermore, we show that pancreatic α-cells produce DA de novo and secrete it. The resulting dopaminergic modulation of pancreatic hormone secretion is dependent upon both cell type- and species-specific differences in expression levels of dopaminergic and adrenergic receptors. Overall, our work demonstrates a key signaling interplay between DA and NE in α- and β-cells as a novel regulatory pathway for pancreatic hormone release and offers the promise of new therapeutic approaches to treat the dysfunctional secretion of insulin and glucagon by targeting peripheral catecholamine receptors.

## Supplementary information

Supplementary Tables

Supplementary Figure S1

Supplementary Figure S2

Supplementary Figure S3
