# Supplementary material for: Dopamine regulates pancreatic glucagon and insulin secretion via adrenergic and dopaminergic receptors
Source: Transl Psychiatry. 2021 Feb 16;11:59. doi: 10.1038/s41398-020-01171-z (PMC7884786; doi:10.1038/s41398-020-01171-z)
Supplement: Supplementary file 1 — Supplementary Tables [file 41398_2020_1171_MOESM1_ESM.docx]

| **Donor** | **Age**  **-Years** | **Disease State** | **Gender** | **BMI** | **COD** | **Pancreas weight (grams)** | **Islet arrival date** |
| --- | --- | --- | --- | --- | --- | --- | --- |
| **1** | 55 | No | F | 22 | Stroke | 118 | 5-10-18 |
| **2** | 45 | No | F | 29.8 | Anoxia | 98.9 | 7-31-18 |
| **3** | 48 | No | M | 24.6 | Anoxia | 84.1 | 2-5-19 |
| **4** | 24 | No | M | 31.74 | Anoxia | 101.8 | 4-5-19 |

**Supplementary Table S1. Human donor demographic information.**

| **Cell Line** | **Clonidine** | **Norepinephrine** | **Dopamine** |
| --- | --- | --- | --- |
| α_2A_-AR KO | N/A | 1.4 ± 0.002 µM | 474 ± 2.5 nM |
| Parental INS-1E | 12.7 ± 1.3 nM | 39.8 ± 1.5 nM | 1.5 ± 0.002 µM |

**Supplementary Table S2. Drug potencies of adrenergic and dopaminergic receptor ligands on glucose-stimulated insulin secretion.** Drug potencies of adrenergic and dopaminergic receptor ligands in inhibition of glucose-stimulated insulin secretion in α_2A_-adrenergic receptor knockout (α_2A_-AR KO) INS-1E cells versus the unmodified parental INS-1E cell line. Values represent mean IC_50_ values ± SEM of n≥3 experiments performed in triplicate.

| **Cell Type** | **Drug** | **K_i_** | **N** |
| --- | --- | --- | --- |
| **INS-1E** | Dopamine | 164 ± 1.2 nM | 4 |
|  | Norepinephrine | 22.5 ± 1.2 nM | 4 |
|  | Clonidine | 0.27 ± 0.001 nM | 4 |
|  | Yohimbine | 92.2 ± 1.1 nM | 3 |

**Supplementary Table S3. Binding parameters (K_i_) at α_2A_-adrenergic receptors determined by competition binding.** Binding parameters from competitive-inhibition experiments of [^3^H]RX821002 versus norepinephrine, dopamine, clonidine, and yohimbine in membrane preparations from unmodified INS-1E cells expressing endogenous α_2A_-adrenergic receptors. K_i_ values are expressed as means ± SEM of n≥3 experiments performed in triplicate.
