# Supplementary material for: Dopamine regulates pancreatic glucagon and insulin secretion via adrenergic and dopaminergic receptors
Source: Transl Psychiatry. 2021 Feb 16;11:59. doi: 10.1038/s41398-020-01171-z (PMC7884786; doi:10.1038/s41398-020-01171-z)
Supplement: Supplementary file 2 — Supplementary Figure S1 [file 41398_2020_1171_MOESM2_ESM.pdf]

a

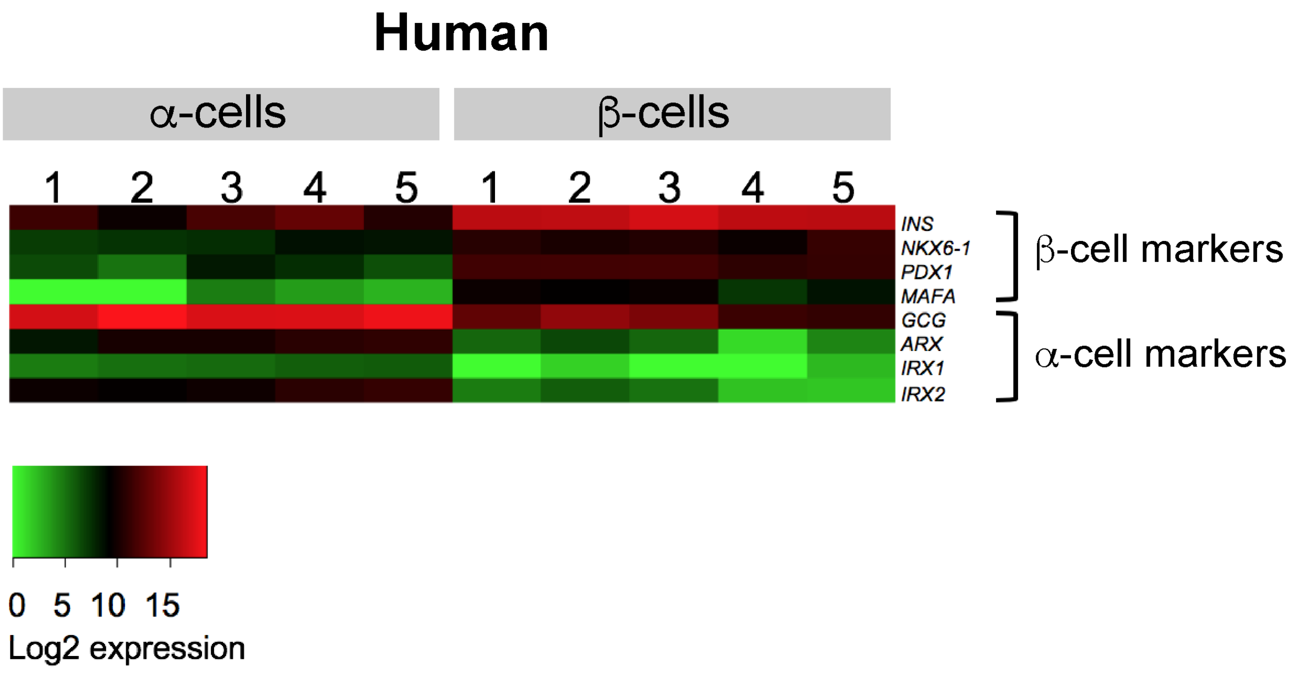

b

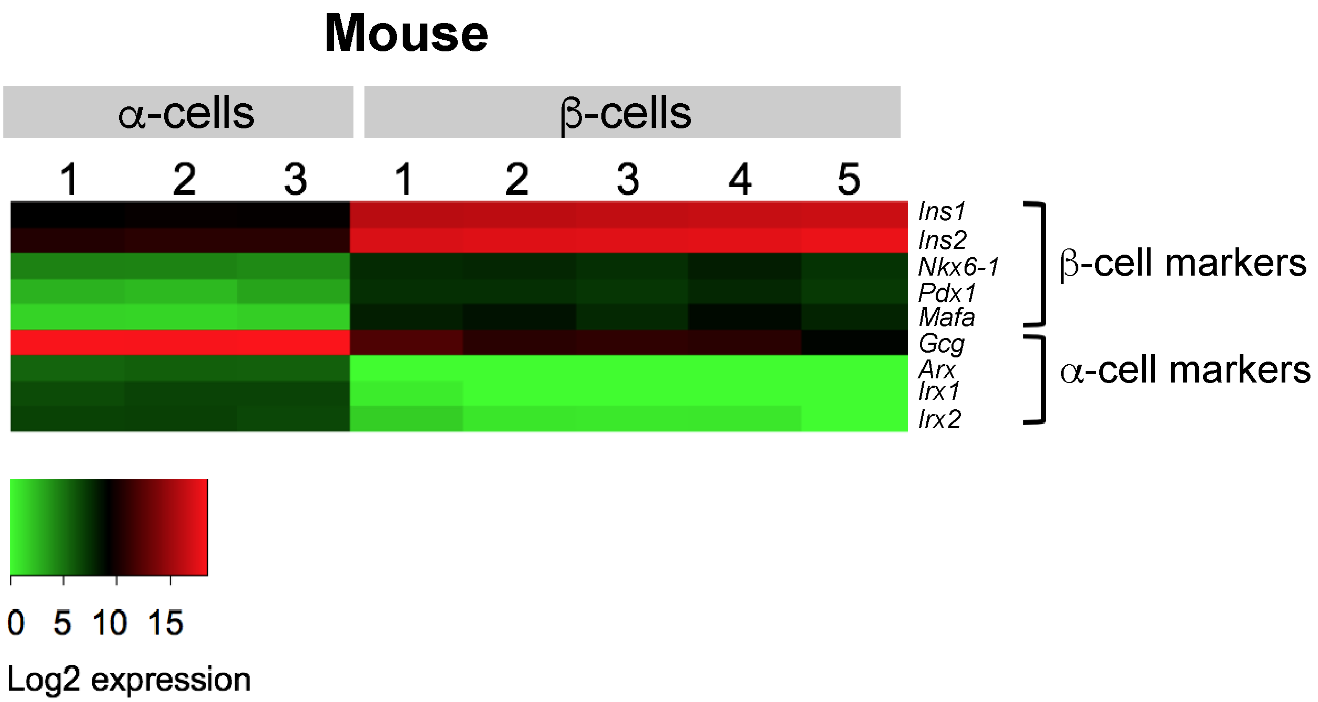

Supplementary Figure S1. Enrichment of cell type-specific markers in human and mouse α- and β-cell RNA-seq datasets. (a, b) Heatmaps of subsets of differentially expressed genes selectively enriched in individual α- and β-cell samples purified from **(a)** human, and **(b)** mouse pancreatic islets.
