# Supplementary material for: Dopamine regulates pancreatic glucagon and insulin secretion via adrenergic and dopaminergic receptors
Source: Transl Psychiatry. 2021 Feb 16;11:59. doi: 10.1038/s41398-020-01171-z (PMC7884786; doi:10.1038/s41398-020-01171-z)
Supplement: Supplementary file 3 — Supplementary Figure S2 [file 41398_2020_1171_MOESM3_ESM.pdf]

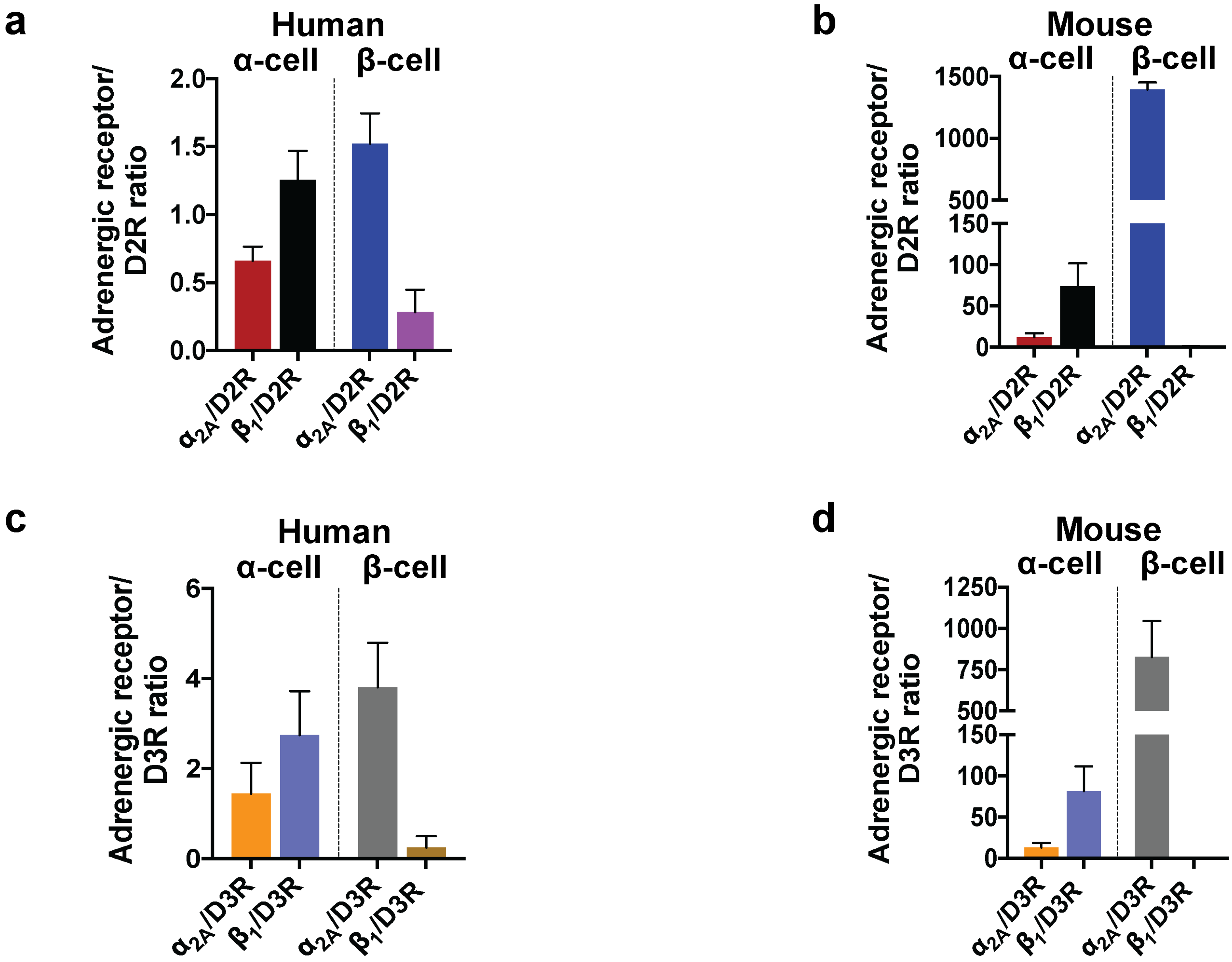

Supplementary Figure S2. Relative ratios of catecholamine receptors expressed in human versus mouse α- and β-cells. Relative ratios comparing expression of α- and β-adrenergic receptors to D_2-like receptors based on RNA-seq datasets from individual α-cell and β-cell samples purified from **(a, c)** human, and **(b, d)** mouse islets. Bar graphs highlight the relative expression differences in adrenergic receptor subtypes compared to D2R or D3R according to species and cell-type. Data are represented as means ± SEM.
