# Supplementary material for: Dopamine regulates pancreatic glucagon and insulin secretion via adrenergic and dopaminergic receptors
Source: Transl Psychiatry. 2021 Feb 16;11:59. doi: 10.1038/s41398-020-01171-z (PMC7884786; doi:10.1038/s41398-020-01171-z)
Supplement: Supplementary file 4 — Supplementary Figure S3 [file 41398_2020_1171_MOESM4_ESM.pdf]

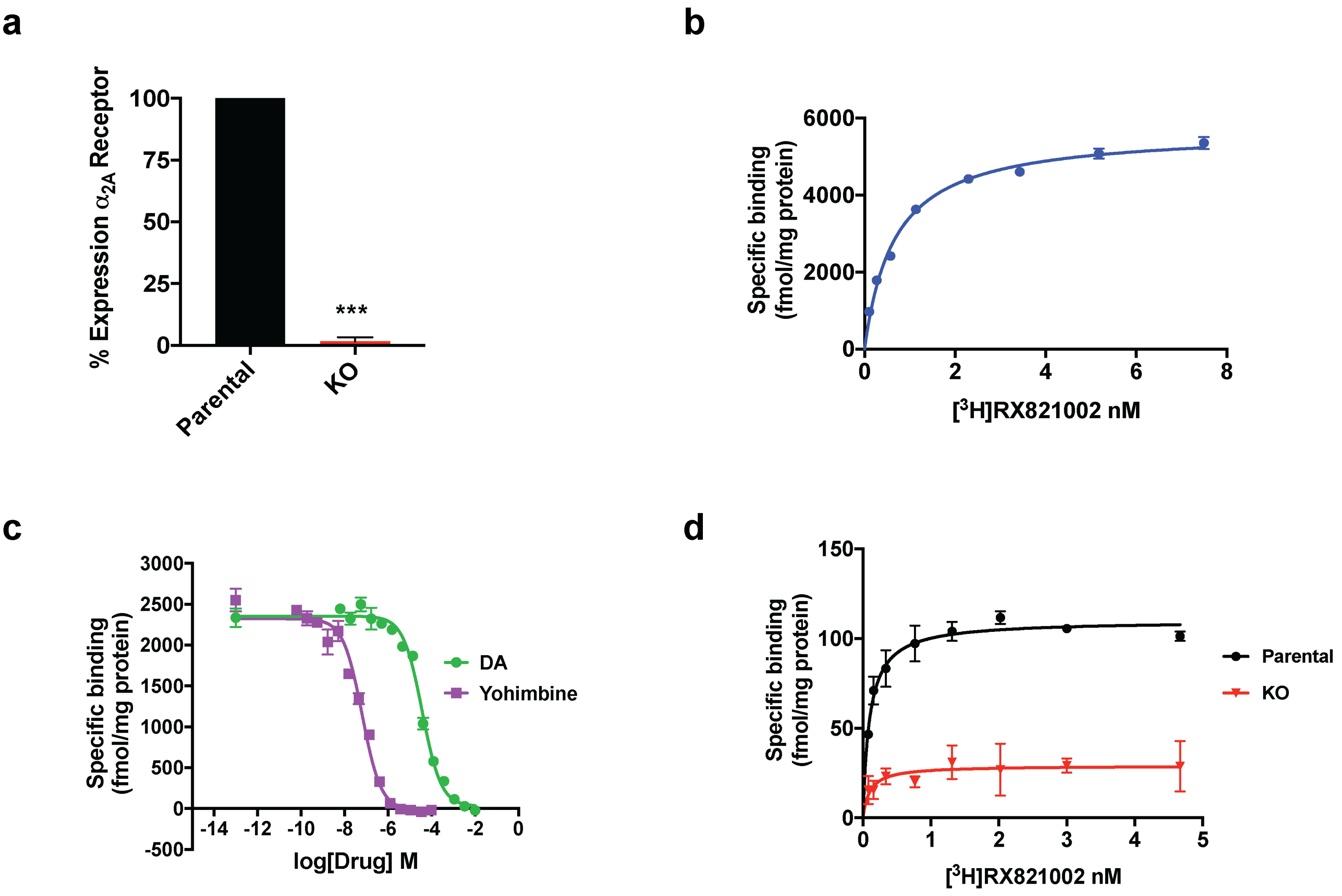

Supplementary Figure S3. Characterization and radioligand binding studies in α_{2A}-adrenergic receptor knockout INS-1E cells. (a) qPCR analysis comparing α_{2A}-adrenergic receptor expression in rat β-cell-derived parental INS-1E cells and the α_{2A}-adrenergic receptor knockout (KO) INS-1E cells. qPCR shows total loss of α_{2A}-adrenergic receptor expression in the KO cells (P=0.0003). Results were normalized to % α_{2A}-adrenergic receptor expression in the unmodified parental INS-1E cells. **(b)** Representative radioligand saturation binding curve with α_{2A}-adrenergic receptor antagonist [^{3}H]RX821002 using membranes prepared from HEK-293 cells transiently overexpressing human α_{2A}-adrenergic receptor (B_{max}=5691±103 fmol·mg^{-1} protein; K_D=0.67±0.05 nM). **(c)** Representative competition curves of [^{3}H]RX821002 versus increasing concentrations of α_{2A}-adrenergic receptor blocker yohimbine (in purple; K_i=38.2±1.1 nM) or DA (in green; K_i=22.1±0.001 M). **(d)** Representative radioligand saturation binding curves comparing [^{3}H]RX821002 binding to endogenously expressed α_{2A}-adrenergic receptor in membranes from α_{2A}-adrenergic receptor KO INS-1E cells (in red) and the unmodified parental INS-1E cell line from which the KO cells were derived (in black; B_{max}=110±0.02 fmol·mg^{-1} protein; K_D=0.098±0.02 nM). Data are represented as means ± SEM and performed in triplicate from n≥3 independent experiments; two-tailed Student's t-test (**a**).
